# Transcriptome-wide association studies identify candidate genes for carcass and meat traits in meat rabbits

**DOI:** 10.3389/fvets.2024.1453196

**Published:** 2024-12-06

**Authors:** Hua He, Xinyang Tian, Zhe Kang, Guozhi Wang, Xianbo Jia, Wenqiang Sun, Song-Jia Lai, Shi-Yi Chen

**Affiliations:** Farm Animal Genetic Resources Exploration and Innovation Key Laboratory of Sichuan Province, Sichuan Agricultural University, Chengdu, Sichuan, China

**Keywords:** TWAS, carcass traits, meat traits, transcriptome, association analysis

## Abstract

Meat rabbits are a small herbivorous livestock and have been popularly raised in China for producing high-quality meat. Therefore, it is economically important to genetically improve both carcass performance and meat quality in meat rabbits. However, we still know less about the underlying candidate genes that may determine phenotypic variation on carcass and meat traits of meat rabbits. The main objective of this study was to identify candidate genes whose mRNA expression levels may be significantly involved in regulating carcass and meat traits of meat rabbits based on the transcriptome-wide association studies (TWAS). Five carcass traits of the carcass weight (CW), dressing out percentage (DP), cut weight of hind legs (LW), weight ratio of cut hind legs to carcass (RLW), and weight of visceral and interscapular fat (WF), as well as two meat traits of the drip loss (DL) and cooking loss (CL) were phenotyped in a F1 crossbred population (*N* = 119) between Zika rabbits and Sichuan White rabbits. The effects of mRNA expression levels of a total of 10,288 genome-wide genes on these seven traits were statistically estimated using the mixed linear model, in which the polygenic background effects were accounted for. Our results revealed two candidate genes (*RDH5* and *MTARC2*) that were statistically significantly associated with LW trait (the adjusted *p* values <0.05), whereas no gene reached the statistically significant threshold for all the remaining six traits. Because of the relatively small sample size analyzed, we alternatively selected 20 candidate genes with the lowest *p* values for every trait and subjected them to functional enrichment analyses, which identified three Gene Ontology (GO) terms that were significantly enriched by the candidate genes of CW and RLW traits. In conclusion, this study used TWAS approach to successfully reveal several candidate genes whose mRNA expression levels may be involved in regulating carcass and meat traits in meat rabbits, which are helpful to explore the underlying molecular mechanism in the future studies.

## Introduction

Domestic rabbits (*Oryctolagus cuniculus*) are a prolific small herbivorous livestock and have been popularly raised for producing meat, as well as wool and fur. China is the largest producer and consumer of rabbit meat around the world (1), with an approximate production of 543 thousand in 2023. Due to high contents of protein, unsaturated fatty acids, and essential amino acids, as well as low fat and cholesterol levels (2, 3), it has been becoming more and more popular to consume rabbit meat and products. In this context, it is desirable to genetically improve both carcass and meat traits in meat rabbits, which have been largely overlooked in comparison to other common livestock, such as pigs (4) and beef cattle (5).

On the whole, the estimated heritabilities were moderate to high for the growth-related traits in meat rabbits, which had been comprehensively reviewed by Blasco et al. (6), García et al. (7), and Helal et al. (8). Many studies have been already conducted to explore the candidate genetic markers and genes that may be significantly associated with growth traits in different breeds of meat rabbits. Gencheva et al. (9) revealed that single-nucleotide polymorphisms (SNPs) of growth hormone and growth hormone receptor genes were significantly associated with individual body weight during the growing periods measured at 35, 70 and 90 days of age in Californian breed rabbits. Using genotyping-by-sequencing technique, genome-wide SNPs were explored among five rabbit breeds, for which the associations were analyzed with various production and morphological traits (10). In contrast, both carcass and meat traits in meat rabbits have been less studied yet. Yang et al. (11) performed the genome-wide association studies (GWAS) of growth, carcass and meat quality traits in meat rabbits, and revealed some candidate SNPs and genes that were significantly associated with some of these traits. Based on GWAS approach, some candidate SNPs and genes were also suggested to be significantly associated with intramuscular fat and fatty acid composition traits in rabbits regarding animal additive genetic effects (12) and maternal genetic effects (13).

One of the foundations of GWAS is dependent on linkage disequilibrium between the genotyped SNPs and causative variants. Therefore, the detection power of GWAS will be influenced by both marker distribution density genotyped and population genomic architecture studied. Another limitation of GWAS is the gaps between the significant variants identified and the finally manifest phenotype, which may be further mediated by some transcriptional regulations. To overcome these limitations, gene expression level-based association analyses, i.e., the transcriptome-wide association studies (TWAS), were suggested to be a promising approach to identify candidate genes that significantly regulate the complex traits (14). Recently, we successfully employed the long-read sequencing technology approach for profiling genome-wide gene expression levels in Longissimus dorsi tissue and subjected them to association analyses with growth traits in meat rabbits (15). In this study, we further investigated candidate genes whose mRNA expression levels are significantly associated with seven carcass and meat traits in meat rabbits, which may be helpful us to better understand the functional genes and pathways that may be involved in regulating these complex traits.

## Materials and methods

### Animals and traits

The animal population was established and first descripted in our previous study (15). In brief, a F1 crossbred population (*N* = 119) between Zika rabbits and Sichuan White rabbits was established in the research farm. All rabbits were weaned and finished at 35 and 84 days of age, respectively. After slaughter, a total of seven carcass and meat traits were measured and collected, including the carcass weight (CW, g), dressing out percentage (DP, %), cut weight of hind legs (LW, g), weight ratio of cut hind legs to carcass (RLW, %), weight of visceral and interscapular fat (WF, g), drip loss (DL, %), and cooking loss (CL, %). The raw phenotypic records were quality-controlled through removing the possible outliers that reside outside the median ± 3.5 × median absolute deviation (16). The definitions of these traits and statistical description are in Table 1.

**Table 1:** Descriptive statistics of the carcass and meat traits analyzed in this study.

Traits	N	Mean	SD	Min	Max	Description
CW (g)	95	917.7	132.5	598.2	1274.2	Weight of skinned, eviscerated, and head-removed carcass.
DP (%)	91	46.2	2.6	38.8	53.3	Ratio of CW to slaughter live weight.
LW (g)	95	340.7	50.0	168.8	430.6	Weight of hind legs cuted at the first sacral vertebra.
RLW (%)	92	37.5	1.5	34.2	42.2	Ratio of LW to CW.
WF (g)	95	9.2	6.1	0.9	23.3	Sum of weights of both visceral and interscapular fat.
DL (%)	95	6.1	2.8	1.4	14.4	Loss of raw meat (L. dorsi) after hung at 4°C for 24 h.
CL (%)	95	65.9	5.7	54.4	79.9	Loss of raw meat (L. dorsi) after cooked at 100°C for 30 min.

CW, carcass weight; DP, dressing out percentage; LW, cut weight of hind legs; RLW, weight ratio of cut hind legs to carcass; WF, weight of visceral and interscapular fat; DL, drip loss; CL, cooking loss. N, number of phenotypic records; SD, standard deviation; Min, minimum value; Max, maximum value.

### Gene expression dataset

The gene mRNA expression dataset was first reported in our previous study (15). In brief, all 119 rabbits were euthanized at 84 days of age and subjected to the collection of Longissimus dorsi tissue. About one μg of the quantified RNA sample was used for Oxford Nanopore single-molecule long-read sequencing. The raw sequencing reads were quality-controlled and normalized across samples using TMM method (17), after which the gene expression levels were quantified and measured as counts per million reads (CPM) using edge R R package v4.0.16 (18). Finally, the mRNA expression dataset consisted of a total of 10,288 genes that were effectively expressed in the Longissimus dorsi tissue among 115 samples (four samples were excluded after quality controls), and the effectively expressed genes were defined if they were present (the raw read count ≥2) within more than 30% of individuals.

### Association analyses and statistical model

According to Mai et al. (19), associations of gene mRNA expression levels with carcass and meat traits were statistically analyzed using the mixed linear model (MLM), which adjusted the polygenic background effects and was defined as:


$$y=w_{i}b_{i}+C\beta +Wu+e$$

where y is the phenotypic value vector of each trait studied; $b_{i}$ is the estimated effect of gene i on the trait with its mRNA expression level vector $w_{i}$ ; C is the incidence matrix of fixed effects, which included the gender (two levels) for LW, RLW, WF, and DL, and gender and birth season (three levels) for CW, DP, and CL, with the effect vector $\vec{\beta }$ . These fixed effects were selected for inclusion in the model based on the backward elimination procedure (*p* < 0.05) of the *lm* function in R software. W is the matrix consisting of the standardized expression levels for all included genes, u is the vector of the polygenic effects of all genes on the trait with $u\sim N\left(0,O\sigma_{o}^{2}\right)$ , in which O is the expression level-based similarity matrix (ORM) and $\sigma_{o}^{2}$ is proportion of phenotype variance explained by all genes; e is the residual effect vector with $e\sim N\left(0,I\sigma_{e}^{2}\right)$ , and the I is an identity matrix. The element of O matrix between individual j and k was computed as (20):


$$O_{jk}=\frac{1}{m}\underset{i}{\sum }\left(x_{ij}-\mu_{i}\right)/\left(x_{ik}-\mu_{i}\right)/\sigma_{i}^{2}\text{,}$$

Where $x_{ij}$ and $x_{ik}$ are the normalized expression levels of gene i in the individual j and k , respectively; $\mu_{i}$ and $\sigma_{i}^{2}$ are the mean and variance of expression level for gene i across all individuals, respectively. In order to avoid the double fitting of one target gene simultaneously considered as both fixed and random effects in the MLM, the MOMENT (multi-component MLM-based omic association excluding the target) module implemented in OSCA software v0.46 was used (20).

### Multiple comparison adjustment and functional analyses

To explore possible issue of population stratification, quantile-quantile (QQ) plots were first visualized according to the raw *p* values obtained for all genes. Second, genomic inflation factor (*λ*) was calculated as the ratio of the median of the observed distribution of $\chi^{2}$ statistic to the expected median, as $\hat{\lambda }=\mathrm{median}\left(\chi^{2}\right)/0.4549$ , for which the 95% confidence interval (CI) was further derived. To address multiple comparison problem, false discovery rate (FDR) approach was used for adjusting the raw *p* values and 0.05 was set to the statistically significant threshold (21). The gene with *p* value lower than this FDR-adjusted threshold was considered to be statistically significant.

In addition to the candidate genes that were revealed to be statistically significant according to the threshold defined, the 20 genes with lowest *p* values were selected for every trait and subjected to functional enrichment analyses. Because of the relatively small sample size included in this study, this more flexible approach may identify the biologically relevant genes, whereas whose *p* values did not reach the defined threshold. The enrichment analyses were conducted using the g:GOSt function of the g:Profiler web server (22), including the target data sets of the Gene Ontology (GO) terms (23) and Kyoto Encyclopedia of Genes and Genomes (KEGG) pathways (24). The default parameters and methods for adjusting for multiple comparison testing (i.e., the build-in g:SCS method) were used, and 0.05 was set to the statistically significant threshold.

## Results

**Figure 1:**
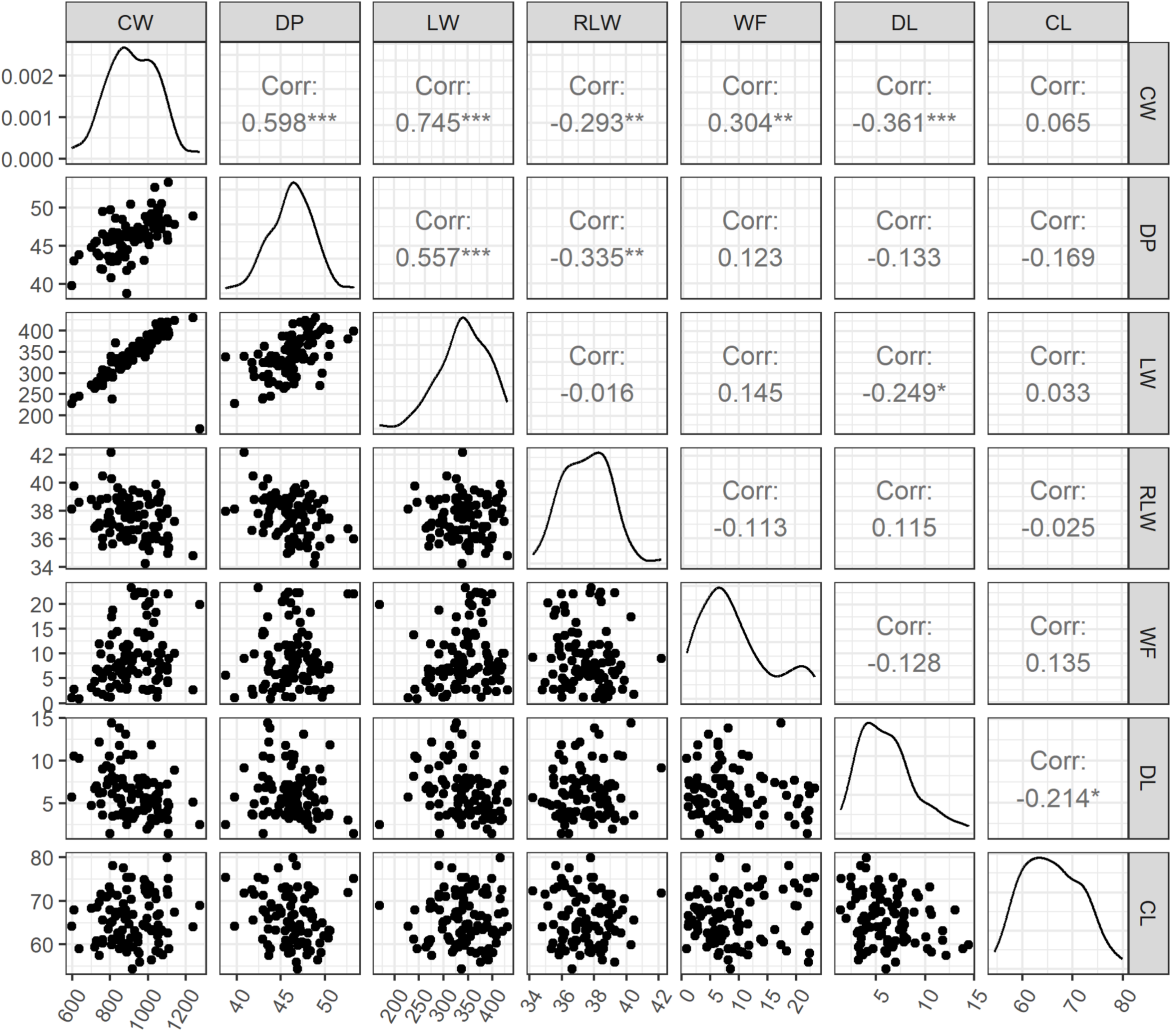
Phenotypic correlations among seven carcass traits and meat traits. CW, carcass weight; DP, dressing out percentage; LW, cut weight of hind legs; RLW, weight ratio of cut hind legs to carcass; WF, weight of visceral and interscapular fat; DL, drip loss; CL, cooking loss. *, **, and *** indicate the statistical significance at 0.05, 0.01, and 0.001, respectively.

The descriptive statistics of all seven traits are in Table 1. The mean (± SD) of CW and DP was 917.7 ± 132.5 g and 46.2 ± 2.6%, respectively. The absolute and relative weight of hind legs was 340.7 g (LW, SD = 50.0 g) and 37.5% (RLW, SD = 1.5%). The WF ranged from 0.9 to 23.3 g, with the mean (± SD) of 9.2 ± 6.1 g. The two meat traits had the means (± SD) of 6.1 ± 2.8% for DL and 65.9 ± 5.7% for CL. The phenotypic correlations were further analyzed among all seven traits (Figure 1). CW had the significant positive correlations with DP (*r* = 0.598, *p* < 0.001), LW (*r* = 0.745, *p* < 0.001), and WF (*r* = 0.304, *p* < 0.01), whereas weak negative correlations were observed with RLW (*r* = −0.293, *p* < 0.01) and DL (*r* = −0.361, *p* < 0.001). There was no significant correlation between LW and RLW. With an exception of CW, WF did not show significant correlation with other five traits. There was weak but significant negative correlation between DL and CL (*r* = −0.214, *p* < 0.05).

**Figure 2:**
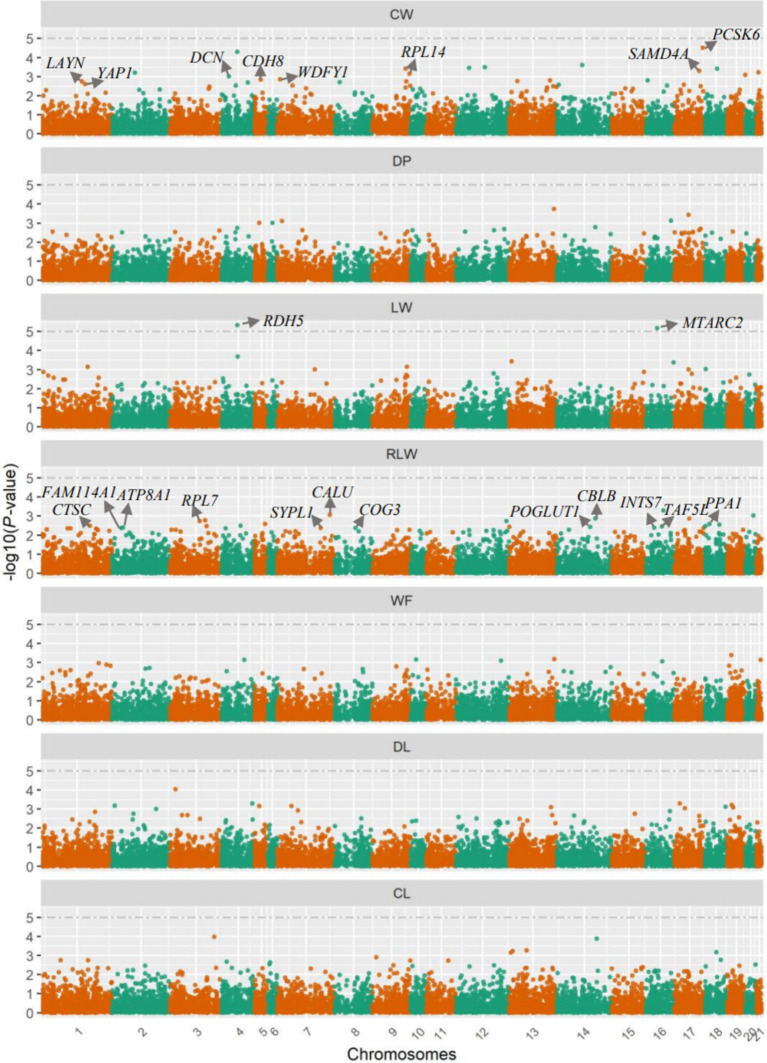
Manhattan plots for the gene expression level-based association analyses. CW, carcass weight; DP, dressing out percentage; LW, cut weight of hind legs; RLW, weight ratio of cut hind legs to carcass; WF, weight of visceral and interscapular fat; DL, drip loss; CL, cooking loss. The significance thresholds are denoted by the dashed lines for every trait. Each point represents each gene, and the significant candidate genes revealed in the present study were further denoted by their gene names.

The association analysis results of gene mRNA expression levels with carcass and meat traits are in Figure 2. Based on the visualization of QQ plots and the quantitative results of *λ* factors that ranged from 0.93 (95% CI: 0.74–1.12) for LW to 1.03 (95% CI: 0.84–1.22) for CW and DP traits (Supplementary Figure S1), it can be concluded that there was no obvious population stratification. According to the statistically significant threshold defined, we identified two protein-encoding genes that were significantly associated with LW trait, including the retinol dehydrogenase 5 (*RDH5*) gene located on Chr4 (*p* = 1.61 × 10^−6^), and the mitochondrial amidoxime reducing component 2 (*MTARC2*) gene located on Chr16 (*p* = 6.80 × 10^−6^). However, no gene reached the statistically significant threshold for other six traits. The lowest *p* values of genes were 3.11 × 10^−5^ for CW, 1.82 × 10^−4^ for DP, 5.02 × 10^−4^ for RLW, 4.08 × 10^−4^ for WF, 9.21 × 10^−5^ for DL, and 1.05 × 10^−4^ for CL, respectively (Supplementary Table S1).

For every trait, we alternatively selected 20 protein-coding genes with the lowest *p* values and subjected them to functional enrichment analyses; the detailed information of these genes, such as raw *p* values and genomic positions, are in Supplementary Table S1. Among them, we revealed two significant GO terms for these candidate genes of CW trait, of CW trait (Table 2), including the molecular function of “glycosaminoglycan binding” (GO:0005539) and cellular component of “cell junction” (GO:0030054), which were significantly enriched by three genes (proprotein convertase subtilisin/kexin type 6, *PCSK6*; layilin, *LAYN*; decorin, *DCN*) and five genes (sterile alpha motif domain containing 4A, *SAMD4A*; ribosomal protein L14, *RPL14*; WD repeat and FYVE domain containing 1, *WDFY1*; cadherin 8, *CDH8*; Yes1 associated transcriptional regulator, *YAP1*), respectively. Furthermore, there were 12 genes significantly enriched into a cellular component of “cytoplasm” for RLW trait, including calumenin (*CALU*), Cbl proto-oncogene B (*CBLB*), ribosomal protein L7 (*RPL7*), inorganic pyrophosphatase 1 (*PPA1*), cathepsin C (*CTSC*), TATA-box binding protein associated factor 5 like (*TAF5L*), protein O-glucosyltransferase 1 (*POGLUT1*), ATPase phospholipid transporting 8A1 (*ATP8A1*), integrator complex subunit 7 (*INTS7*), component of oligomeric golgi complex 3 (*COG3*), family with sequence similarity 114 member A1 (*FAM114A1*), and synaptophysin like 1 (*SYPL1*). There was no significantly enriched biological function for other five traits, and no KEGG was significantly enriched for any of all traits analyzed.

**Table 2:** The significantly enriched GO terms for the 20 candidate genes with the lowest *p* values for every trait.

Trait	Term name	Term ID	*p*	Genes
CW	glycosaminoglycan binding	GO:0005539	0.013	*PCSK6, LAYN, DCN*
CW	cell junction	GO:0030054	0.049	*SAMD4A, RPL14, WDFY1, CDH8, YAP1*
RLW	cytoplasm	GO:0005737	0.020	*CALU, CBLB, RPL7, PPA1, CTSC, TAF5L, POGLUT1, ATP8A1, INTS7, COG3, FAM114A1, SYPL1*

CW, carcass weight; RLW, weight ratio of cut hind legs to carcass; *p*, the adjusted *p* values.

## Discussion

A large number of GWAS have been successfully used for identifying causative genetic variants and genes that are significantly associated with diverse production traits in all kinds of livestock (25). However, there are some gaps from GWAS results to biological insights. Despite of the improved detection power of gene expression level-based association analysis as illustrated in human genetic studies (26), TWAS approach has not been widely employed yet in livestock mainly because of the relatively high cost on quantifying transcriptomic expression in a large-scale sample. Based on the imputed gene expression data, Chhotaray et al. (27) used the TWAS approach for identifying significant causative loci for milk production and its composition in 136 Murrah buffaloes. In Huaxi cattle, both GWAS and TWAS approaches were integrated to reveal several candidate genes that are significantly associated with the weight of Longissimus dorsi muscle (28). We recently profiled the transcriptomic landscape using long-read RNA sequencing technology and successfully performed TWAS for several growth traits of meat rabbits (15). In the present study, we similarly used TWAS approach for further revealing the candidate genes for carcass and meat traits in meat rabbits.

In meat rabbits, carcass performance has the important economic values. The differential carcass traits were previously reported between two rabbit genotypes (29). Based on 186 microsatellite and three SNP markers, a highly significant quantitative trait loci (QTL) located on Chr7 was revealed to be associated with carcass weights in rabbits (30). Using GWAS approach, Yang et al. (11) identified tens of genome-wide SNPs that are significantly associated with the carcass traits in rabbits, and suggested the most possible causative gene of lipase A, lysosomal acid type (*LIPA*) that encodes the lipase A, the lysosomal acid lipase (cholesterol ester hydrolase). In the present study, our TWAS did not identify any gene that is statistically significantly associated with the carcass weight. However, two functional GO terms were significantly enriched among the eight candidate genes that had the relatively low *p* values. In both of them, the GO term of “glycosaminoglycan binding” was previously suggested to be involved in the regulation of water holding capacity in Chinese Simmental beef cattle (31). The involved *DCN* gene that encodes a member of the small leucine-rich proteoglycan family of proteins was also found to be differentially expressed in vitamin A-restricted Japanese Black steers (32). Furthermore, *SAMD4A* and *CDH8* genes found in this study were previously suggested to be candidate genes associated with drip loss (33) and carcass traits (34) in pigs, respectively.

We found in the present study that CW had the moderate and high positive correlations with DP and LW. However, there was no gene or functional gene set revealed to be significantly associated with DP, for which several candidate genes were previously reported (11). Regarding the important carcass cut trait of LW in meat rabbits (35), we identified two statistically significant genes of *RDH5* and *MTARC2*. *MARC2* is involved in adipogenesis and regulated by nutritional status (36). The knock-out mice of *MARC2* were resistant to high-fat diet-induced obesity through being responsible for *N*-reductive biotransformation (37). *MTARC2* was also reported to be a candidate gene significantly associated with the fatness in pigs (38) and meat performance in quails (39). *RDH5* is involved in the retinol metabolism pathways, and the increased retinol metabolism promotes lipolysis in adipose tissue (40). Based on copy number variations, *RDH5* was previously suggested to be associated with feed conversion rate in Nellore cattle (41). In this context, we suggested that both *RDH5* and *MTARC2* may be highly possibly involved in the regulation on muscle growth in meat rabbits, which can be subjected to selection with aiming to genetically improve the carcass or meat quality traits. Here, we did not detect genes that are significantly associated with the simple measure of WF trait in meat rabbits, whereas there were studies that previously suggested a common genetic regulation between fat deposition in muscle and carcass performance (42). Furthermore, we cannot exclude possible association of *RDH5* and *MTARC2* genes with other intramuscular fat traits.

Based on the GWAS, several genes, such as EWS RNA binding protein 1 (*EWSR1*), apolipoprotein L domain containing 1 (*APOLD1*), and phospholipase B domain containing 1 (*PLBD1*), were suggested to be significantly associated with intramuscular fat traits in the divergently selected lines of rabbits (43). Similarly, 15 genome-wide significant SNPs were detected to affect feed conversion rate and residual feed intake in rabbits (44). The genetic polymorphisms of five genes of insulin-like growth factor-binding proteins (*IGFBPs*) were scanned in four rabbit breeds and revealed to significantly affect the acidity, color, and shear force traits of rabbit meat (45). For the two meat traits analyzed in the present study, we revealed that CW has a weak negative correlation with DL, which is favorable as the individuals that have a greater carcass weight will have the decreased drip loss. Similarly, no significant gene or functional gene set was identified in the present study to affect the two meat traits.

There are two practical implications regarding the results obtained in the present study. First, it revealed several candidate genes of carcass and meat traits in meat rabbits based on the association analyses of gene mRNA expression level, which will facilitate the follow-up studies to dissect underlying molecular regulation mechanism. Second, the candidate genes identified in the present study can be included for optimizing the genomic evaluation models, such as through assigning the greater weights to the genetic variants located in or tightly linked to these candidate genes. On the other hand, we acknowledged that one limitation of this study is the relatively small sample size in comparison with the ordinary GWAS, which will decrease the detection power. Another limitation is that only two simple traits of meat quality were phenotyped, and more desirable meat characteristics, such as the intramuscular fatty acid contents and composition traits, remain to be investigated.

## Conclusion

In this study, we measured seven carcass and meat traits in meat rabbits and conducted the association analyses using transcriptomic data. Several candidate genes, such as *RDH5* and *MTARC2*, were suggested to be significantly associated with some of these traits. The results are helpful to explore underlying molecular mechanism and optimize genomic evaluation models to genetically improve the carcass performance and meat quality in meat rabbits.

## Data Availability

The original contributions presented in the study are publicly available. This raw 295 sequencing reads can be found here: https://ngdc.cncb.ac.cn/gsa/; CRA013219. The 296 normalized and quantified gene expression data can be found here: 297 https://doi.org/10.6084/m9.figshare.27804465.v1.
